# Pedicle screw insertion into infected vertebrae reduces operative time and range of fixation in minimally invasive posterior fixation for thoracolumbar pyogenic spondylitis: a multicenter retrospective cohort study

**DOI:** 10.1186/s12891-024-07565-0

**Published:** 2024-06-10

**Authors:** Hisanori Gamada, Toru Funayama, Yusuke Setojima, Keigo Nagasawa, Takane Nakagawa, Kotaro Sakashita, Shun Okuwaki, Kaishi Ogawa, Shigeo Izawa, Yosuke Shibao, Hiroshi Kumagai, Katsuya Nagashima, Kengo Fujii, Yosuke Takeuchi, Masaki Tatsumura, Itsuo Shiina, Masafumi Uesugi, Masashi Yamazaki, Masao Koda

**Affiliations:** 1 Department of Orthopaedic Surgery, Institute of Medicine, University of Tsukuba, 1-1-1 Tennodai, Tsukuba, Ibaraki 305-8575 Japan; 2 Department of Orthopaedic Surgery, Ibaraki Western Medical Center, Chikusei, Ibaraki Japan; 3 Department of Orthopaedic Surgery, Kenpoku Medical Center, Takahagi Kyodo Hospital, Takahagi, Ibaraki Japan; 4 Department of Orthopaedic Surgery, Showa General Hospital, Kodaira, Tokyo Japan; 5 Department of Orthopaedic Surgery, Moriya Daiichi General Hospital, Moriya, Ibaraki Japan; 6 Department of Orthopaedic Surgery, Ichihara Hospital, Tsukuba, Ibaraki Japan; 7 Department of Orthopaedic Surgery, Tsukuba Central Hospital, Ushiku, Ibaraki Japan; 8 Department of Orthopaedic Surgery and Sports Medicine, Tsukuba University Hospital, Mito Clinical Education and Training Center, Mito Kyodo General Hospital, Mito, Ibaraki Japan; 9 Department of Orthopaedic Surgery, Ibaraki Seinan Medical Center Hospital, Sakai, Ibaraki Japan

**Keywords:** Minimally invasive spine surgery, Pedicle screws, Posterior fixation, Pyogenic spondylitis

## Abstract

**Background:**

Minimally invasive posterior fixation surgery for pyogenic spondylitis is known to reduce invasiveness and complication rates; however, the outcomes of concomitant insertion of pedicle screws (PS) into the infected vertebrae via the posterior approach are undetermined. This study aimed to assess the safety and efficacy of PS insertion into infected vertebrae in minimally invasive posterior fixation for thoracolumbar pyogenic spondylitis.

**Methods:**

This multicenter retrospective cohort study included 70 patients undergoing minimally invasive posterior fixation for thoracolumbar pyogenic spondylitis across nine institutions. Patients were categorized into insertion and skip groups based on PS insertion into infected vertebrae, and surgical data and postoperative outcomes, particularly unplanned reoperations due to complications, were compared.

**Results:**

The mean age of the 70 patients was 72.8 years. The insertion group (*n* = 36) had shorter operative times (146 versus 195 min, *p* = 0.032) and a reduced range of fixation (5.4 versus 6.9 vertebrae, *p* = 0.0009) compared to the skip group (*n* = 34). Unplanned reoperations occurred in 24% (*n* = 17) due to surgical site infections (SSI) or implant failure; the incidence was comparable between the groups. Poor infection control necessitating additional anterior surgery was reported in four patients in the skip group.

**Conclusions:**

PS insertion into infected vertebrae during minimally invasive posterior fixation reduces the operative time and range of fixation without increasing the occurrence of unplanned reoperations due to SSI or implant failure. Judicious PS insertion in patients with minimal bone destruction in thoracolumbar pyogenic spondylitis can minimize surgical invasiveness.

## Background

Pyogenic spondylitis is an infectious disease with an annually increasing prevalence in an aging society [1–3]. While the gold standard for treatment involves antimicrobial agents and conservative therapy, including rest [4], recent advancements in surgical techniques and instruments have allowed the widespread use of minimally invasive surgery for early intervention [5–7]. Among these methods, posterior fixation, particularly using percutaneous pedicle screws (PPS), is preferred because of its minimal invasiveness and association with fewer postoperative complications [8–10]. 

Historically, metallic implant insertion was contraindicated for infected sites. Instead, the conventional approach involved thorough debridement of the abscess and infected vertebral bodies, followed by autogenous bone grafting. Lately, advancements in surgical techniques and infection control have allowed the insertion of metallic cages into infected intervertebral spaces and pedicle screws (PS) into infected vertebrae [11–13]. Nevertheless, the safety and efficacy of PS insertion into infected vertebrae via posterior fixation procedures remain controversial. There is a paucity of literature regarding the safety of this method and its impact on infection control; also, the prevalence of the most concerning surgical site infections (SSI) remains unclear [13]. 

Thus, we aimed to evaluate the safety and efficacy of PS insertion into infected vertebrae via minimally invasive posterior fixation for thoracolumbar pyogenic spondylitis.

## Methods

The protocol for this multicenter retrospective cohort study was approved by the local institutional review board. We hypothesized that, provided there is no abscess formation, PS insertion into infected vertebrae in pyogenic spondylitis does not increase postoperative complications and improves treatment outcomes.

For this, we retrospectively reviewed a consecutive series of 100 patients who underwent minimally invasive posterior fixation procedures, mainly using PPS, for thoracolumbar pyogenic spondylitis refractory to conservative therapy. Based on the methodology of previous studies, the patients across nine affiliated institutions were followed-up postoperatively for at least 6 months since January 2014 [9,14]. Cases in which planned anterior or posterior debridement, bone grafting, or similar procedures were performed, were excluded [9]. 

We evaluated the patients’ surgical records and categorized each patient into two groups based on the presence or absence of PS insertion into the infected vertebrae, i.e., the upper and lower vertebrae of the infected intervertebral level [13].

**Fig. 1:**
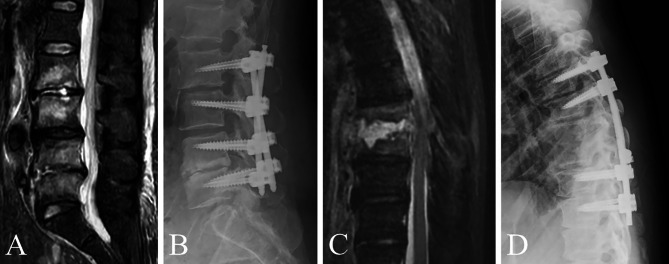
Illustrative cases of the insertion (**A** and **B**) and skip (**C** and **D**) groups (**A** and **B**): Posterior fixation was performed from L2 to L5, including PS insertion into the infected vertebrae for pyogenic spondylitis at L2/3 and L4/5. (**C** and **D**): Posterior fixation was performed from T6 to T11 for pyogenic spondylitis at T8/9 by skipping T8 and T9 vertebrae

1. Insertion group (Fig. 1a and b): At least one PS was inserted into the infected vertebrae, including cases where PSs were inserted into all pedicles and those where the trajectory was absent due to bone destruction, resulting in partial insertion.

2. Skip group (Fig. 1c and d): No PS was inserted into the infected vertebrae.

**Fig. 2:**
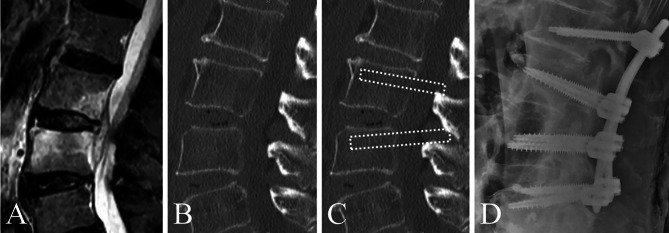
Criteria for pedicle screw insertion in infected vertebrae in the insertion group. (**A**) L3/4 pyogenic spondylitis. (**B**) On the computed tomography, bone destruction was localized to the endplate. (**C**) Since there was no bone destruction along the trajectory of the pedicle screw (dashed line), the decision was made to insert the screw (**D**)

**Fig. 3:**
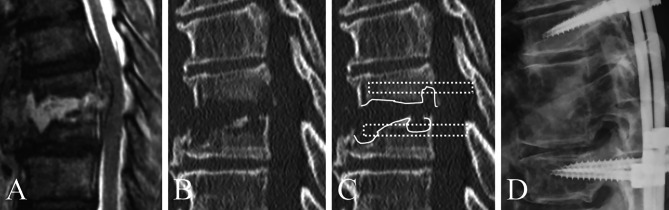
Criteria for pedicle screw insertion in infected vertebrae in the skip group. (**A**) T8/9 pyogenic spondylitis. (**B**) Computed tomography scan showing significant bone destruction in the vertebral body involving the endplate. (**C**) Due to the significant bone destruction (solid line) along the trajectory of the pedicle screw (dashed line), the decision was made to skip the screw (**D**)

The decision for inserting a PS into the infected vertebra was based on whether the planned PS trajectory was subject to bone destruction due to infection on computed tomography (CT); the final decision was at the discretion of the operating surgeon in each institution (Figs. 2 and 3).

### Outcome measures

Data concerning the following variables were recorded for each patient:

- Demographic and clinical factors: sex, age, region of the spine affected (thoracic, lumbar, thoracolumbar, or lumbosacral), number of infected intervertebral spaces, comorbidities (diabetes, cancer, chronic kidney disease requiring hemodialysis, cirrhosis, and others), presence of extravertebral abscess (epidural, iliopsoas, empyema), bone destruction of the infected vertebrae, and causative organism.
- Surgical data: operative time, estimated blood loss, and range of fixation.
- Postoperative outcomes: postoperative follow-up period, instances of unplanned reoperation due to postoperative complications and the corresponding reasons (especially SSI), implant failure, and need for additional surgery due to poor infection control.

According to a previous study, bone destruction of the infected vertebrae on preoperative CT scan was graded as 0 (the vertebral body endplate having almost a normal appearance), grade 1 (irregular vertebral endplate, defined as bone destruction < 3 mm from the height of the endplate), grade 2 (bone destruction > 3 mm but not extending to the posterior wall), and grade 3 (bone destruction > 3 mm extending to the posterior wall) [15]. In cases where multiple disk levels were infected, the grade of the infected vertebrae with the greatest degree of bone destruction was selected. The term “range of fixation” was defined as the number of vertebrae included in the fixation, starting from the most proximal fixed vertebra to the most distal one, including skipped vertebrae where screws were not inserted.

### Statistical analysis

The following variables were compared between the insertion and skip groups: age, postoperative follow-up, number of infected intervertebral spaces, operative time, estimated blood loss, and range of fixation. Continuous variables were compared using Welch’s t-test and nominal variables were evaluated using Fisher’s exact test or chi-square test and via residual analysis (as applicable). A p-value of < 0.05 was considered statistically significant. We used JMP software (version 10; SAS Inc., Cary, NC, USA) for all analyses.

## Results

### Demographic and clinical factors

The final analysis involved 70 patients with a mean age of 72.8 years. Three patients who underwent planned debridement and bone grafting and 27 patients with short follow-up were excluded. The insertion group comprised 36 patients (20 patients with screws inserted in all infected vertebrae and 16 patients with partial insertion), whereas the skip group comprised 34 patients. The mean postoperative follow-up time was 25.4 months (standard deviation, SD = 16.5; range = 6–71 months). The lumbar spine was the most affected spinal location, with single-level infection being the most common variety. The distribution of the grade of bone destruction of the infected vertebrae significantly differed between the two groups (*p* = 0.0080). Specifically, the insertion group had a higher proportion of patients with grade 1 bone destruction (19 vs. 5, *p* = 0.0014) and a lower proportion of patients with grade 2 bone destruction (13 vs. 22, *p* = 0.023) than the skip group based on the residual analysis. There were no significant differences in other patient characteristics between the two groups (Table 1).

**Table 1:** Demographic and clinical characteristics of the patients (*N* = 70)

	Insertion Group (*n* = 36)	Skip Group (*n* = 34)	Adjusted residual	*p*-value
Sex (n)				0.80^a^
Male	24	24		
Female	12	10		
Age (mean, years)	73.9	71.6		0.35^b^
Postoperative follow-up period (mean, months)	22.7	28.4		0.15^b^
Location				0.56^c^
Lumbar	23	16		
Thoracic	9	13		
Thoracolumbar	4	4		
Lumbosacral	1	1		
Number of infected intervertebral levels (mean)	1.28	1.21		0.61^b^
1	28	29		
2	6	4		
3	2	0		
4	0	1		
Comorbidity (including duplicated)	35	28		0.052^a^
Diabetes	16	8		
Cancer	6	11		
Daily steroid use	4	2		
Hemodialysis due to chronic renal failure	2	0		
Liver cirrhosis	1	0		
Others	13	17		
Extravertebral abscess (including duplicated)	25	24		1.00^a^
Epidural abscess	22	19		
Iliopsoas abscess	15	11		
Empyema	1	1		
Bone destruction of the infected vertebral disk				0.0080^c^
Grade 0	0	1	−1.04	0.23
Grade 1	19	5	3.35	0.0014
Grade 2	13	22	−2.39	0.023
Grade 3	4	6	−0.78	0.29

^a^Fisher’s exact test; ^b^Welch’s t-test; ^c^chi-squared test

The specified rate of the causative organism was 71% (*n* = 50/70 patients), with the *Streptococcus species* being the most common. There was no difference in the specified rate of causative organisms between the two groups (Table 2).

**Table 2:** Distribution of the causative organisms of pyogenic spondylitis among the participants (*N* = 70)

Organisms	Insertion Group (*n* = 36)	Skip Group (*n* = 34)	*p*-value
Specified	27	23	0.60a
* Streptococcus species*	11	8	
MSSA	7	3	
* Escherichia coli*	0	9	
* Klebsiella pneumoniae*	4	0	
MRSA	1	0	
MRSE	0	1	
Others	4	2	
Not specified	9	11	

^a^Fisher’s exact test. MSSA: Methicillin-susceptible *Staphylococcus aureus*, MRSA: Methicillin-resistant *Staphylococcus aureus*, MRSE: Methicillin-resistant *Staphylococcus epidermidis*

### Surgical data

The mean values for the operation time and the range of fixation were smaller in the insertion group compared to the skip group (146 min versus 195 min, *p* = 0.032; 5.4 vertebrae versus 6.9 vertebrae, *p* = 0.0009, respectively) (Table 3).

**Table 3:** Surgical outcomes of the patients (*N* = 70)

	Insertion Group (*n* = 36)	Skip Group (*n* = 34)	*p* value^a^
Operation time (min)	146	195	0.032
Estimated blood loss (g)	122	253	0.10
Range of fixation (number of fixed vertebrae)	5.4	6.9	0.0009

^a^Welch’s t-test

### Unplanned reoperations due to postoperative complications

The incidence of unplanned reoperations was 24% (*n* = 17/70) in the study cohort. Reasons for unplanned reoperation included SSI, implant failure, and poor infection control in 4 (5.7%) patients, residual abscess in 2 (2.9%) patients, and other reasons in 3 (4.3%) patients, including vertebroplasty for proximal or distal junctional failure (*n* = 2) and decompression for postoperative neurological symptoms (*n* = 1) (Table 4).

**Table 4:** Distribution of the incidence of unplanned reoperations for postoperative complications

	Insertion Group (*n* = 36)	Skip Group (*n* = 34)	*p* value^a^
Causes of unplanned reoperations	8	9	0.78
Surgical site infection	2	2	1.00
Implant failure	2	2	1.00
Poor infection control of pyogenic spondylitis	0	4	0.051
Residual abscess	2	0	0.49
Others	2	1	
No reoperation	28	25	

^a^Fisher’s exact test

**Fig. 4:**
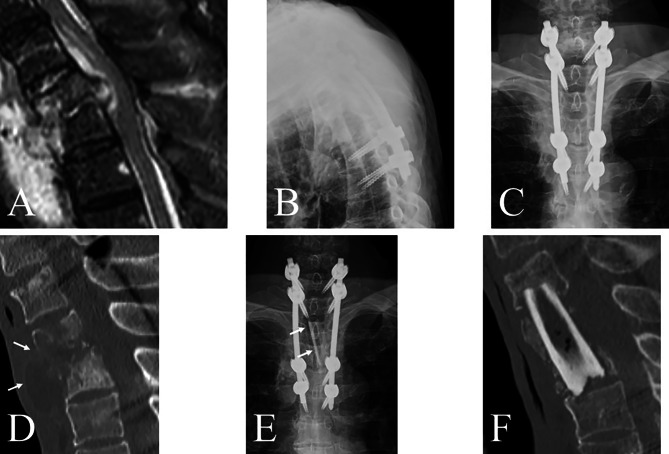
Illustrative case of unplanned reoperations in pyogenic spondylitis due to poor infection control. (**A**) T2/3 pyogenic spondylitis with an epidural abscess. (**B** and **C**) Despite skipping T2 and T3 vertebrae and performing posterior fixation from C7 to T5, infection control was not achieved, and an abscess remained anterior to the vertebral bodies (**D** arrows). Subsequently, anterior debridement and autogenous bone grafting from the fibula (**E** arrows and **F**) were performed, resulting in successful infection control

The incidence of unplanned reoperations was comparable in both groups (*p* = 0.78). However, unplanned reoperations due to poor control of pyogenic spondylitis were only required in the skip group; all patients underwent anterior surgical debridement and autogenous bone grafting (Fig. 4). The causative organism in four patients with poor infection control was identified. Among these patients, one and three presented with grade 0 and 2 bone destruction, respectively.

## Discussion

The key findings of this study were:

1. PS insertion into infected vertebrae during minimally invasive posterior fixation for thoracolumbar pyogenic spondylitis allowed for shorter operative times and a smaller range of fixation compared to those managed without PSs.
2. There was no increase in unplanned additional surgeries, including SSI, associated with metal insertion into infected vertebrae.

The existing literature contains limited evidence supporting the use of PS insertion in infected vertebrae [13]. A single-center retrospective study reported that skipping PS into infected vertebrae led to an enlarged range of fixation and a greater need for additional surgery due to pseudoarthrosis [13]. There is a lack of uniform criteria guiding the use of PS insertion into infected vertebrae; some studies on posterior fixation for pyogenic spondylitis report that PS insertion into infected vertebrae is often skipped in cases with prominent bone destruction, whereas others report that it can be performed where there is mild bone destruction [8–10,13,16]. In surgical fixation of vertebral fractures, PS insertion into the fractured vertebra is preferred because it allows enhanced stability and maintenance of vertebral height and alignment, reduces the need for additional surgeries, and has no adverse effects on bone fusion [17–19]. While PS insertion into infected vertebrae in pyogenic spondylitis is believed to improve local stability and contribute to better outcomes, the existing research is contentious about its utility and safety.

The present study demonstrated that PS insertion into infected vertebrae does not increase the incidence of adverse events, particularly SSI. Moreover, the insertion group had a smaller range of fixation and operative time compared with the skip group, upholding the minimal invasiveness of the posterior fixation approach. We included a relatively large sample size (*n* = 70) from multiple health facilities, which is a substantial number in the context of surgical management of pyogenic spondylitis.

PS insertion into infected vertebrae offers the advantage of reducing surgical invasiveness, particularly in terms of the range of fixation and operative time. This is especially beneficial for elderly patients with an average age of 72.8 years and comorbidities, where curtailing the invasiveness of surgical procedures becomes crucial. Previous reports consistently indicate that skipping screws results in a broader fixation range, which is consistent with our findings [13]. 

It is well-documented that the insertion of instrumentation, including PS, for fixation in pyogenic spondylitis stabilizes the infectious focus and helps in infection control [8–13,16]. While the insertion of the metal itself is not considered hazardous, concerns about SSI have traditionally been reported at approximately 6.7–17%, with notably higher rates (60%) for methicillin-resistant *Staphylococcus aureus* [20–22], which is similar to that observed in our study. In this study, the decision on whether to insert PS into the affected vertebra was determined using preoperative CT. This was done to ensure that the PS is not inserted into prominent infection foci, such as a zone of significant bone destruction.

Four patients in the skip group required additional anterior surgery because of poor infection control, whereas two patients in the insertion group had residual abscesses. In various reports, the incidence of additional anterior surgery following inadequate infection control with isolated posterior fixation in the surgical treatment of pyogenic spondylitis is reported to range from 2–17%.^[6, 9, 16, 23–24^ The skip group had a higher proportion of patients with strong bone destruction of the infected vertebrae than the insertion group. Severe bone destruction of the infected vertebrae is a predictor of resistance to conservative treatment and the need for surgery [15]. Regardless of PS insertion into the infected vertebra, there are instances of inadequate infection control with posterior fixation alone. Notably, our results highlight that not all patients can be treated with minimally invasive posterior fixation alone, necessitating anterior surgery for those with prominent bone destruction or drainage in case of residual abscesses.

This study has certain limitations. First, we used a retrospective study design. Although criteria for PS insertion into affected vertebrae were established in this multicenter study, real-time evaluation was left to the discretion of the treating physician at each facility, introducing potential variability. In addition, differences in patient history in PS insertion criteria included mild bone destruction in the insertion group and more extensive bone destruction in the skip group. The difference in the degree of bone destruction caused selection bias. In addition, three cases were excluded where preoperative bone destruction was extensive and the surgeon deemed additional anterior surgery necessary, further introducing selection bias. Future studies should include a higher number of cases and must utilize techniques, such as propensity score matching, to align patient history, such as the extent of bone destruction, between groups. This approach can help accurately examine the efficacy of PS insertion into the affected vertebrae, or determine cases that require anterior surgery.

Nevertheless, this multicenter investigation involved patient data from various health facilities, including an university hospital, general hospitals, and those located in urban and rural areas; hence, the findings are generally representative of this disease population. However, caution is advised when extrapolating the results to regions with different age demographics or variations as this study was conducted in a developed country where most population is elderly with comorbidities. Further prospective studies are warranted to determine the effect of PS insertion into infected vertebrae on postoperative outcomes.

## Conclusions

PS insertion into infected vertebrae in minimally invasive posterior fixation for thoracolumbar pyogenic spondylitis reduces operative time and range of fixation without increasing the incidence of unplanned reoperations due to SSI or implant failure. Furthermore, surgical invasiveness can be minimized by inserting PS into infected vertebrae in patients with minimal bone destruction.

## Data Availability

The datasets generated during and analyzed during the current study are available from the corresponding author upon reasonable request.

## Abbreviations

PS: Pedicle screws
PPS: Percutaneous pedicle screws
SSI: Surgical site infections
CT: Computed tomography
SD: Standard deviation
